# Endovascular Double-Layer Bare Stent Placement in the Treatment of Posttraumatic Pseudoaneurysm

**DOI:** 10.1155/2021/5575173

**Published:** 2021-03-11

**Authors:** Wenle Tan, Shubin Dou, Jijin Yang, Jiang Xiong, Xiquan Zhang, Feng Duan

**Affiliations:** ^1^Department of Interventional Radiology, The General Hospital of Chinese People's Liberation Army, Beijing 100853, China; ^2^Traumatic Interventional Department, The 960th Hospital of Chinese People's Liberation Army, Zibo, Shandong Province 255300, China; ^3^Department of Interventional Radiology, Changhai Hospital, Shanghai 200433, China; ^4^Department of Vascular Surgery, The General Hospital of Chinese People's Liberation Army, Beijing 100853, China

## Abstract

**Objective:**

To investigate the efficacy and safety of endovascular double-layer bare stent placement for the treatment of traumatic false aneurysm (TFA).

**Methods:**

This is a retrospective review of five patients with TFA undergone double-layer bare stent placement in our center between February 2011 and August 2020. There are 2 males and 3 females aged 29-65 years, with an average age of 43 years. One case suffered from common carotid artery pseudoaneurysm, and four cases suffered superficial femoral artery pseudoaneurysm.

**Results:**

The endovascular interventional treatment was successful in all 5 patients, and the pseudoaneurysms disappeared after treatment. No TFA recurrence and no complications such as instent stenosis, stent migration, stent fracture, endoleak, and infection were observed during the 3-99-month follow-up period.

**Conclusion:**

For the treatment of TFA, endovascular interventional therapy with double-layer bare stent was minimally invasive, safe, and effective with fewer complications. It could preserve all branches of parent artery and had the advantage of lower cost. It can be used in the treatment of TFA in selected cases. However, further clinical researches with larger cohorts are needed before its long-term efficacy can be completely clarified.

## 1. Introduction

Traumatic false aneurysm (TFA) refers to peripheral hematoma caused by the partial rupture of the arterial vascular wall after injury. Since the vascular lumen is connected to the hematoma, a tumor-like expansion of the hematoma cavity may occur under the high arterial blood pressure. The lesion is known as a pseudoaneurysm because there is no normal three-layer structure of arteries [1]. Pseudoaneurysm can, if left untreated, become a life-threatening situation by thrombosis, rupture, or distal embolization [2]. Thus, proper and timely treatment is very important.

Before the era of endovascular techniques, open surgical aneurysmectomy was the conventional treatment for TFA [3]. In recent years, with the advances of new tools and embolization materials, endovascular interventional therapy, a minimally invasive and safe method, has been applied by interventional radiologists to treat TFAs, giving satisfactory results [4]. Based on the locations, types, and collateral pathways of pseudoaneurysm, different individualized treatments should be adopted, including single or overlapped stent-assisted coiling and covered stents [5, 6]. Zhang et al. reported that multiple overlapping uncovered stents could be a feasible option in the endovascular management of complicated peripheral and visceral artery aneurysms where side branches need to be maintained [7]. There was no similar case in the literature using dual-bare stents for the treatment of TFA with a long-term follow-up for 99 months. This study is aimed at evaluating the long-term clinical therapeutic effect of double-layer bare stent implantation for TFA.

## 2. Subjects and Methods

### 2.1. General Data

In this study, five TFA patients with an average age of 43 years (2 males and 3 females, aged 29-65 years) were admitted to our emergency department from February 2011 to August 2020. The causes of TFA included sharp injury in 1 case and traffic injuries in 4 cases. The TFA injury sites included common carotid artery (*n* = 1) and superficial femoral artery (*n* = 4). The clinical manifestations consisted of continuously enlarging pulsatile mass in the site of injury, palpable tremor, vascular murmur, and discomfort and pain in the limbs on the traumatic side. The diagnosis was confirmed by computed tomography angiography (CTA) before operation.

### 2.2. Treatment

All five patients underwent CTA before treatment to determine the exact location, diameter, morphology, and collateral circulation of TFA [8]. After preoperative and intraoperative examination and assessment, double-layer bare stent placement was performed in all patients.

#### 2.2.1. Surgical Procedure

Five milliliters of 2% lidocaine hydrochloride was subcutaneously injected for local anesthesia. After intravenous administration of heparin sodium (0.6-0.8 mg/kg), the femoral artery was punctured using the standard Seldinger technique. With the help of supersmooth guide wire, the catheter was delivered to the proximal vessel of TFA. Digital subtraction angiography (DSA) was performed to determine the location, morphology and size of TFA, the diameters of the distal and proximal blood vessels, and the main branch vessels that maybe involved. The tip of the catheter was inserted into the normal blood vessel to establish a working channel for the guide wire. The stent was slowly released after accurate positioning with the aid of bony landmarks and DSA path diagram. Since the diseased segment involved important collateral circulation, overlapping bare stents were placed. At least 2 cm of anchoring area was required at the two ends of pseudoaneurysm. The bare stents (LifeStent, Bard Company, USA) were slowly placed across the neck of the pseudoaneurysm, and the therapeutic effect was evaluated by DSA. If there was no endoleak or if the blood flow into the hematoma cavity slowed down significantly, the implantation of bare stents was stopped; if there was still contrast agent filling in the hematoma cavity, another bare stent was implanted [9].

#### 2.2.2. Criteria for Successful Treatment of TFA with Endovascular Stent Implantation

The final angiography showed good stent position and an immediate decrease of flow in the sac of pseudoaneurysm. All branches of the parent artery were preserved. The endovascular stents implantation was regarded as successful. After the operation, the femoral artery puncture point was sutured with a conventional vascular suture device to stop bleeding, followed by local compression for about 10 minutes. Subsequently, the femoral artery puncture point was compressed with elastic bandage for 24 hours.

### 2.3. Follow-Up

CTA or angiography was performed 3, 6, and 12 months after discharge and then annually to observe if there was recurrence of TFA, the blood flow of collateral vessels, and the possible migration, rupture, and stenosis of stents, endoleak, and infection.

## 3. Results

### 3.1. Therapeutic Efficacy

Double-layer bare stents (LifeStent, Bard Company, USA) were successfully implanted in all five patients. Postoperative angiography showed that the pseudoaneurysm was hardly visible, and no important collateral vessels were occluded. All patients experienced local mass shrinkage and pain relief, and the pulsation and arterial murmur disappeared. The ischemic symptoms were improved. No wound infection was found, and there was no interventional therapy-associated complication. The overall therapeutic efficacy was satisfactory.

### 3.2. Follow-Up Outcome

The postoperative follow-up lasted 3 to 99 months (median: 24 months). All patients showed good outcome during the follow-up period, and no complications such as instent stenosis, migration, rupture, or endoleak were observed.

### 3.3. Typical Cases

**Figure 1:**
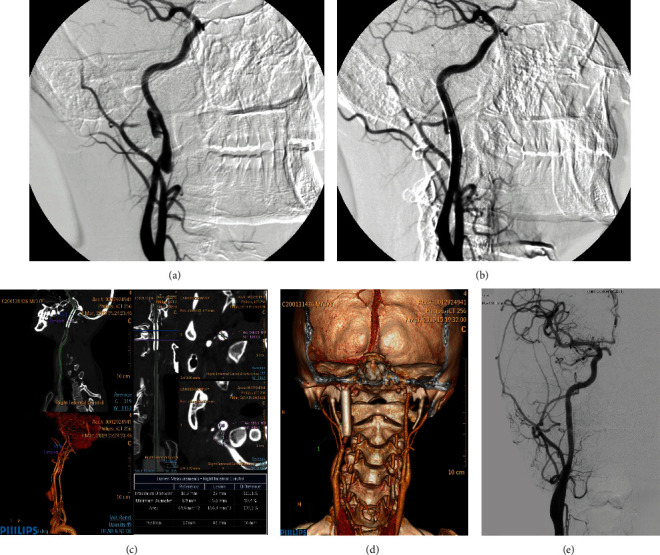
Pseudoaneurysm of the right common carotid artery caused by head trauma. (a) DSA before operation in February 2011 revealed a pseudoaneurysm of the right common carotid artery. (b) After double-layer bare stent placement, DSA showed that the hematoma cavity disappeared and no contrast extravasation. (c, d) CTA in Mar 2019 revealed that the blood flow in the stent lumen was smooth, and there was no stenosis in the lumen. (e) Angiography in May 2019 showed that the blood flow in the stent lumen was smooth, and there was no stenosis in the lumen.

**Figure 2:**
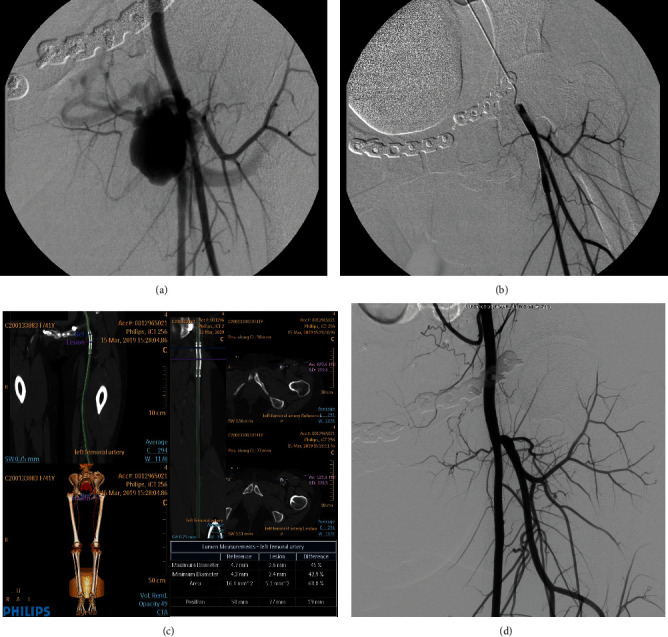
A pseudoaneurysm of the left superficial femoral artery due to a comminuted pelvic fracture. (a) DSA before the operation in March 2014 revealed a pseudoaneurysm at the root of the left superficial femoral artery with a 2.9 × 2.2 cm of maximum diameter. (b) After double-layer bare stent placement, DSA showed that the hematoma cavity disappeared and no contrast extravasation. (c) CTA in Mar 2019 revealed that the blood flow in the stent lumen was smooth, and there was no stenosis in the lumen. (d) Angiography in May 2019 revealed that the blood flow in the stent lumen was smooth, and there was no stenosis in the lumen.

1. Case 1. A 29-year-old male patient with pseudoaneurysm of the right common carotid artery caused by head trauma (Figure 1)
2. Case 2. A 36-year-old female patient presented with a pseudoaneurysm of the left superficial femoral artery due to a comminuted pelvic fracture (Figure 2)

## 4. Discussion

TFA is mostly caused by direct or indirect violence to the arteries. Since the pseudoaneurysm is composed of fibrous tissue, it cannot heal naturally. Most TFAs increase in size gradually with the impact of blood flow. TFA may rupture and bleed easily without proper and timely treatment. And once the thrombus on the inner wall of the blood vessel falls off, it may cause distal arterial embolism which compresses the veins and nerve adjacent and causes serious outcomes, even death [10]. The current therapy of TFA includes conservative treatment, surgical treatment, and endovascular treatment. With the development of interventional technology, endovascular minimally invasive treatment has largely replaced surgical treatment. It mainly includes endovascular covered stent-graft exclusion, implantation of multiple layer overlapping bare stents, and stent-assisted coil embolization [11, 12]. However, stent-assisted coiling has the risk of follow-up recurrence and rerupture [13]. The covered stent showed poor compliance and could lead to endoleak without complete apposition and occlude the arterial branches [14]. Aleksandar et al. reported a case of a giant hepatic artery aneurysm treated with dual layer stent placement as flow-diverting option. The hypothesis of preserving side branches that arise from the aneurysm or close to it may be an additional potential advantage of dual layer stents over traditional stent grafts [15].

With regard to selection of stents for endovascular interventional therapy in TFA patients, multiple factors should be considered, such as long-term patency, risk of vascular rupture, coexisting thrombosis, and surrounding collateral arteries. Treatment should be individualized for different patients. The covered stent can directly cover the rupture or distal end of the pseudoaneurysm through a physical barrier, which separates the blood stream from the TFA cavity and thus prevent blood flow from impacting the hematoma cavity. As the pressure in the hematoma cavity decreases, emboli form inside the hematoma and gradually occlude the pseudoaneurysm [16]. However, the covered stent has disadvantages of affecting collateral occlusion and poor compliance. Multiple overlapping bare stents can keep important collateral vessels patent and thus are safe and effective in treating TFA [17], with relatively lower cost. In this study, all the five patients underwent double-layer bare stent implantation, and the clinical symptoms of these patients were significantly improved without obvious complications. During the long-term follow-up (up to 99 months), CTA revealed that the hematoma cavity disappeared, and the artery where the pseudoaneurysm was located had smooth blood flow. No obvious stenosis in the stent was observed. Therefore, it is concluded that double-layer bare stent placement is one of the effective methods for TFA.

Our study was limited by its single-arm retrospective design and the small sample size. Thus, we cannot make the conclusion that double-layer bare stents can replace covered stents in the treatment of pseudoaneurysm. However, with the accumulation of cases and the extension of follow-up time, double-layer bare stent placement may become an alternative to covered stent, especially for cases with important collateral circulation. Further investigations are necessary to define the feasibility, safety, efficacy, and durability associated with this treatment option.

## Data Availability

The data used to support the findings of this study are included within the article.
